# Risk Assessment in Monitoring of Water Analysis of a Brazilian River

**DOI:** 10.3390/molecules27113628

**Published:** 2022-06-06

**Authors:** Luciene Pires Brandão, Vanilson Fragoso Silva, Marcelo Bassi, Elcio Cruz de Oliveira

**Affiliations:** 1 Postgraduate Programme in Metrology, Pontifical Catholic University of Rio de Janeiro, Rio de Janeiro 22451-900, Brazil; lucienepb27@gmail.com; 2 Veiga de Almeida University, Rio de Janeiro 20271-020, Brazil; vanilson@sigconsultoria.com.br; 3 SIG Consultoria e Assessoria Ltda, Rio de Janeiro 22745-004, Brazil; 4 Telos Soluções Ambientais, São Paulo 13049-322, Brazil; marcelobassicosta@gmail.com; 5 Logistics, Operational Planning and Control, Measurement and Product Inventory Management, PETROBRAS S.A., Rio de Janeiro 20231-030, Brazil

**Keywords:** biochemical oxygen demand, manganese molar concentration, guard bands, pH, non-parametric methods, *Escherichia coli*

## Abstract

This study aimed to introduce non-parametric tests and guard bands to assess the compliance of some river water properties with Brazilian environmental regulations. Due to the heterogeneity of the measurands pH, Biochemical Oxygen Demand (BOD), manganese molar concentration, and *Escherichia coli*, which could be wrongly treated as outliers, as well as the non-Gaussian data, robust methods were used to calculate the measurement uncertainty. Next, based on guard bands, the compliance assessment was evaluated using this previous uncertainty information. For these four measurands, partial overlaps between their uncertainties and the specification limit could generate doubts about compliance. The non-parametric approach for calculating the uncertainty connected to the guard bands concept classified pH and BOD as “conform”, with a risk to the consumer of up to 4.0% and 4.9%, respectively; in contrast, manganese molar concentration and *Escherichia coli* were “not conform”, with a risk to the consumer of up to 25% and 7.4%, respectively. The methodology proposed was satisfactory because it considered the natural heterogeneity of data with non-Gaussian behavior instead of wrongly excluding outliers. In an unprecedented way, two connected statistical approaches shed light on the measurement uncertainty in compliance assessment of water analysis.

## 1. Introduction

Due to the increase of the world population and the unrestrained occupation of industry and housing on riverside lands, a severe problem has arisen in the environment: river pollution [1]. The three significant sources of pollution can be considered as derived from industry (chemical waste products that are discharged into rivers), agriculture (fertilizers and pesticides), and domestic activity (people without environmental awareness that throw rubbish into rivers or from cities without proper sanitary wastewater treatment) [2].

Monitoring and evaluating the quality of surface and groundwater are key factors for the proper management of water resources [3]. The characterization and analysis of trends in watersheds are essential for various management activities, such as planning, granting, charging, and framing of watercourses [4].

The concern with river water analysis is worldwide and can be highlighted in the most varied aspects, such as evaluation of fecal coliform (FC) concentration in river water in Kyrgyzstan, Central Asia [5]; development of a new ensemble machine-learning model for predicting monthly water quality at the Lam Tsuen River in Hong Kong [6]; application of soft computing to predict water quality in wetland [7]; exploitation of the water quality of the Surma River by applying hydrochemical and multivariate statistical methods and also with the help of Water Quality Index (WQI) analysis [8]; spatial and seasonal evaluation of the variations of surface water quality in the reservoir using multivariate statistical techniques along with the Trophic State Index and Trophic State Index deviation [9]; assessment of water quality in the polluted stretch using a cluster analysis during pre- and COVID-19 lockdown of the Tawi River Basin, Jammu, North India [10]; performance of an ecotoxicological evaluation of treated wastewater and river water in the catchment scale using a battery of biotests in Poland [11]; optimal operation of an Iranian surface water resources system in terms of quantity and quality simultaneously by using a Non-Dominated Sorting Genetic Algorithm-II (NSGA-II) algorithm [12]; evaluation and classification of the surface water quality in Dong Thap province, Vietnam, using set-pair analysis and national WQI methods [13]; use of radical terraces for erosion control and water quality improvement in Rwanda [14]; detection of phthalates in drinking and river water samples [15]; evaluation of the suitability of an Arabian surface water [16].

Specifically, concerning Brazilian rivers, some recent studies show the importance of this monitoring and evaluation, such as the use of complex network modeling to evaluate the influence of the parameters on the water quality of rivers in the interior of São Paulo State [17]; the variation of water quality in a hydrographic basin in Paraná [18]; the monitoring and modeling for management of the Piabanha River rehabilitation [19]; the development and application of a new method applied to river samples from a populated tropical urban area [20]; the evaluation of the influence of the water quality seasonality in a neotropical river in Bahia [21]; the evaluation of the influence of the land on the variability of water quality in a State of Sergipe river basin [22]; the development and optimization of an alternative analytical method using an ultrasonic bath to extract metals from Doce River sediment samples in the State of Minas Gerais [23].

The water analysis assessment in Brazilian rivers is based on the maximum allowable limits established by the Brazilian National Environmental Council (CONAMA) Resolution 357/2005 [24]. However, not always; it is a trivial task since the temporal heterogeneity of the samples must be treated instead of considering them as outliers. This issue can be potentialized when there is a partial overlap between the most probable value accompanied with its uncertainty and the specification limit, which could generate doubts about compliance with the specification. To solve this problem, this study aimed to innovatively introduce non-parametric tests connected to guard bands to assess the conformity of river water analysis concerning Brazilian environmental regulations.

## 2. Materials and Methods

This study shows the importance of monitoring the analyzed biochemical parameters in water analyses and describes how to connect robust methods and use uncertainty information in compliance assessment. This section describes an overview of the importance of quality control and how the analysis of each biochemical parameter is described.

### 2.1. Biochemical Parameters

This section describes an overview of the importance of quality control and how the analysis of each biochemical parameter is described.

#### 2.1.1. pH

Measurement by pH is significant for evaluating the environmental conditions of water collections. There is a range of pH values suitable to lifeforms, but it is usually considered a good water quality indicator if pH lies between 5 and 9 [25].

The samples were collected and analyzed in situ within two hours, using proper pH meter equipment, i.e., a meter that was able to perform the measurements in accordance with the reference [26]. The calibration was based on the supplier orientation by means of an indicating (glass) electrode and a reference electrode using National Institute of Standards and Technology (NIST) buffers.

#### 2.1.2. Biochemical Oxygen Demand (BOD)

Biochemical oxygen demand (BOD) testing determines the relative oxygen requirements of wastewaters, effluents, and polluted waters. This parameter is related to water quality and is widely used to evaluate the environmental health of natural water collections, such as rivers and lakes. It is also important in assessing the performance of wastewater treatment facilities. The standard method recommends filling an airtight bottle of the particularized size with the sample until it overflows and incubating it at the specified temperature (in the dark at 20 °C) and time (5 days). BOD was calculated based on the difference between the initial and final measurements of dissolved oxygen [27]. This Standard Method recommended using the dilution technique for higher concentrations in more polluted water or industrial wastewater.

#### 2.1.3. Manganese molar concentration

Manganese is a common element on Earth’s crust, occurring in the form of mineral. It is the twelfth most abundant element and the fourth most abundant metal for commercial use. The average manganese content in the Earth’s crust is around 0.1 %, and in groundwaters, it is less than 0.1 mg L^−1^. Manganese generally is present in iron minerals due to its chemical similarity and is found in oceans, fresh waters, and soils. In aqueous matrices, the common aqueous species are Mn^2+^ and Mn^4+^. Raised manganese levels can cause stains in domestic utensils, but on the other hand, this metal is very useful for plant and animal survival. The United Nations Food and Agriculture Organization considers that in irrigation waters, its concentration cannot exceed 0.2 mg L^−1^.

The analytical method used is Inductively Coupled Plasma-Mass Spectrometry (ICP-MS). This method is based on the measurement of ions generated by a radiofrequency inductively coupled plasma. After being nebulized inside the equipment, chemical species are transported as an aerosol by an argon flow that reached the plasma torch. After this, the ions produced are appropriately introduced into a mass spectrometer [28].

#### 2.1.4. *Escherichia coli*

Detection and evaluation of coliforms are usual indicators of a water’s quality and suitability for domestic, industrial, or other uses. *Escherichia coli* is used as an indicator of environmental contamination in freshwater systems since this organism is rife in human and animal excrements and not generally found in other ecosystems. *Escherichia coli* could be easily detected by its ability to ferment glucose, and it may be easier isolated than other known gastrointestinal pathogens.

The samples collected for this work were submitted to enzyme–substrate tests that use hydrolyzable chromogenic and fluorogenic substrates for simultaneously detecting enzymes produced by total coliforms and *Escherichia coli*. In this method, total coliform bacteria produce the enzyme β-d-galactosidase. Once this enzyme is released in a medium containing chromogenic substrate, this substrate is cleaved to release chromogen. Conversely, most *Escherichia coli* strains produce the other similar enzyme, the β-glucuronidase, which cleaves a fluorogenic substrate in the medium to release fluorogen. Therefore, by observing whether chromogen or fluorogen is released, it is possible to confirm the presence of *Escherichia coli*. The release of chromogen indicates that coliform bacteria are present, and the release of fluorogen indicates that *Escherichia coli* are present [29].

In this study, the *Escherichia coli* density was reported as a most probable number (MPN), based upon a serial dilution of the test sample.

### 2.2. Robust Statistical Methods

Robust or non-parametric statistical methods can be used to describe position and precision measures of results that deviate from normal behavior, as they do not always require the treatment of outliers. In general, robust methods should be used in preference to methods that exclude results wrongly considered as outliers [30].

The median (Med), the scaled median absolute deviation (MADe), and the normalized interquartile range (nIQR) are allowed as simple estimators for data sets with normal behavior and that depart from normality. In these cases, Med, MADe, and nIQR have a greater variance than the mean and standard deviation when applied to data with approximately normal distribution; that is, a more conservative approach [31]. However, these estimators can be used with robustness in both situations. Furthermore, the literature emphasizes that different conclusions can be reached by choosing between MADe, Equation (1) and nIQR, Equation (2) [32].

(1) $$\mathrm{MADe}\left(x\right)=s^{*}\mathrm{MADe}=1.483\;\mathrm{Med}\left|x_{i}-\mathrm{Med}\left(x\right)\right|$$

(2) $$\mathrm{nIQR}\left(x\right)=s^{*}\mathrm{nIQR}=0.7413\times \left(q_{3}-q_{1}\right)$$

where *q_1_* and *q_3_* are the first and third quartiles, respectively.

The value to be evaluated, $\left(x^{*}\right)$, came from a robust statistic calculated using median and robust standard deviation, s*, Equations (1) and (2). For *p* observations, the robust standard uncertainty of this value, evaluation of type A, $u\left(x^{*}\right)$, could be estimated by Equation (3):

(3) $$u\left(x^{*}\right)=1.25\times \frac{s^{*}}{\sqrt{p}}$$

Based on ISO 13528 [31], when using Equation (3), the median value and the robust standard deviation, Equations (1) and (2) were determined from several samples; the uncertainty of median value could be assumed to include the effects of uncertainty concerning heterogeneity. The factor 1.25 (Equation (3)) was based on the variability of the median as a trial to estimate the mean, and it is recommended when testing results have a non-Gaussian behavior. Finally, the expanded uncertainty, $U\left(x^{*}\right),$ was calculated by multiplying $u\left(x^{*}\right)$ by the coverage factor (*k*) for a confidence level of 95%.

### 2.3. Use of Uncertainty Information in Compliance Assessment

Conformity assessment can be understood as any process that evaluates if standards and specified requirements are met. In conformity assessment, a measurement result accompanied with its uncertainty can be useful to decide whether an item of interest meets a requirement. This requirement can be evaluated based on one or two tolerance limits [33].

Once the acceptance range of permissible measured values of a measurand is defined, the risks of false acceptance/rejection decisions related to measurement uncertainty can reach a balance to minimize the costs associated with these improper decisions.

Typical scenarios arise when measurement results accompanied by their measurement uncertainties are used to assess compliance with an upper specification limit. When the measured value plus its uncertainty is above or below the control limit is clear. However, in other situations, false decisions can be made due to the partial overlap of the uncertainty bands about the specification limits [34].

**Figure 1:**
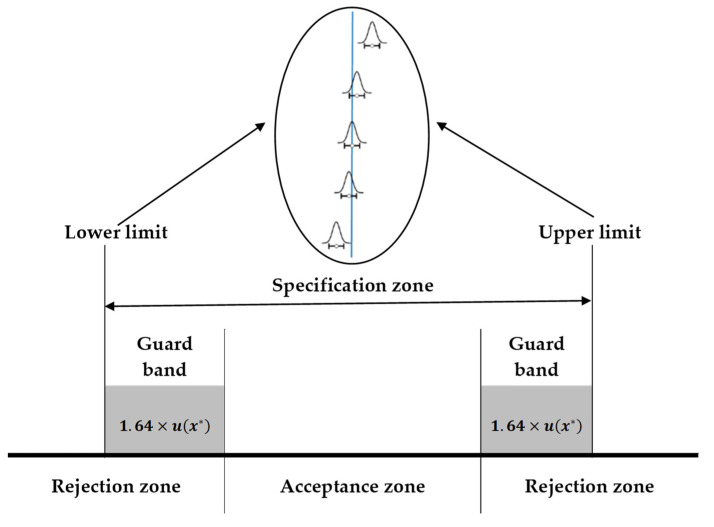
Acceptance and rejection zones for simultaneous upper and lower limits based on guard bands.

A good and current alternative for conformity assessment is the concept of decision rules based on guard bands that are based on measurement uncertainty. Based on this premise, a rejection zone can be outlined as specification limit *L* plus a value *g* (called the guard band). This amount was selected in such a way that for a measurement result equal to or greater than *L + g*, the false rejection probability is less than or equal to *α*; that is, a low probability that the allowable limit has not been exceeded. Figure 1 illustrates the consumer’s risk, which can be defined as the probability of a false acceptance (i.e., accepting an item or lot outside the specification limits).

An excellent option for applying this approach is the Monte Carlo method (MCM). In a simple way, the basis of Monte Carlo simulations is to randomly select a number with a known probability distribution function and repeat this routine *n* times [35], using the median and the robust standard uncertainty. Subsequently, histograms can represent graphically this probability density function, and the guard bands help us to define if the item of interest meets a specified requirement. Finally, Bayes’ theorem can be used to estimate the risk of the consumer [36].

The use of uncertainty information in compliance assessment has been published in original research and studies in areas covering the latest in basic and applied research in analytical sciences related to the risk of false conformity assessment applied to automotive fuel analysis employing a multiparameter approach [37]; connected to a data reconciliation approach solving the discrepancy between producers’ and consumers’ measurements [38]; to study the probability of false conforming in customs control of denatured alcohols [39]; to shed light on the importance of analytical chemistry concerning industrial practices [40]; applied to drug and medicine analyses [41]; use of measurement uncertainty in the conformity/non-conformity assessment of pharmaceutical products [42]. Finally, it is worth highlighting an innovative study that discussed the environmental behavior concerning measurement uncertainty of concentrations of pollutants using the guard bands approach [43].

## 3. Experimental

**Figure 2:**
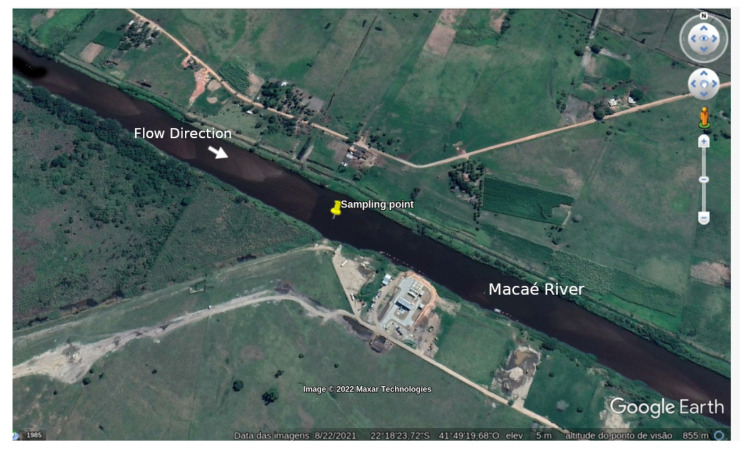
Macaé River.

To characterize the compliance assessment of the water analysis, samples were collected at the same locations, varying over time, at coordinates S 22°18′23.4″, W 41°49′21.5″, in 2020 from January to December, on the right margin of Macaé River, Brazil, Figure 2. The Macaé River basin is about 1710 km^2^, covering six municipalities in Rio de Janeiro state [44]. In the 1940s, the analyzed section of the Macaé River was rectified to facilitate the drainage of swamps in the region. This process changed the behavior of the river, its depth, and the speed of its waters.

The sampling and analytical strategy is based on the environmental monitoring of the Macaé River for use in a thermal power plant electrical system. The number of analyses was 52 for pH (every seven days), 94 for BDO (every four days), 72 (every five days) for Manganese molar concentration, and 60 for *Escherichia coli* (every six days). All the samples were collected at the same point by a system of stainless-steel bucket, separated into proper sample bottles, and sent to the laboratory in a thermal shipping box.

Every sample was labeled and sent to a laboratory except pH measurements, which were analyzed in situ using a Sanxin SX836 portable pH meter, in accordance with the preservation procedures for each parameter. The samples from the Macaé River Margin were carried to the laboratory in ice-cooled boxes to avoid degradation. After standard preservation procedures, the samples were sent to an accredited laboratory for the BOD, Mn, and *Escherichia coli* measurements following the selected measurement procedure for each measurand by ISO/IEC 17025 [45], located in Rio de Janeiro City, 190 km from the sample point. This laboratory has procedures to monitor the validity of results, such as the use of certified reference materials, use of control charts, participation in intralaboratory and interlaboratory comparisons, etc.

## 4. Results

The measurement uncertainty is considered as the square root of the quadratic sum of the analytical uncertainty and sampling uncertainty. The approach used in this study considered both the analytical variability and the temporal variability, without stratifying each uncertainty source. The raw data (before any statistical treatment) are available in Table 1.

### 4.1. Preliminary Statistical Evaluation

Data from Table 1 were treated, and they do not follow a normal distribution based on the Kolmogorov–Smirnov test; in addition, several outliers are met [46].

### 4.2. Statistical Treatment

Here, Equations (1)–(3) were applied to all data from Table 1 without excluding any outliers since this study has considered that the variability of the results is due to the temporal heterogeneity of the sampling target. The results of the statistical treatment are presented in Table 2. In a traditional approach, i.e., always considering that data are normally distributed, and there are no outliers, U(x) SD is calculated $\mathrm{as}\;k\times \mathrm{SD}/\sqrt{p}$, where SD is the standard deviation. There are partial overlaps between the central measures (medians) plus expanded uncertainties and their respective specification limits. It can generate doubts concerning the compliance assessment.

### 4.3. Compliance Assessment

The uncertainty is used to evaluate the conformity/non-conformity with the specification. In this study, the value of g is 1.64 $u\left(x^{*}\right)$ for a significance level of 5%, where $u\left(x^{*}\right)$ is calculated from Equation (3).

Next, histograms with the central value (median of actual data distribution without excluding outliers), its respective uncertainty, i.e., guard band (1.64 $u\left(x^{*}\right))$ from Equation (3), and specification limit(s) are presented. In this study, Monte Carlo simulations were carried out with 100000 pseudorandom values for each water analysis parameter. Here, the consumer’s risk is evaluated, i.e., the risk of a false acceptance of the analyzed item.

#### 4.3.1. pH

**Figure 3:**
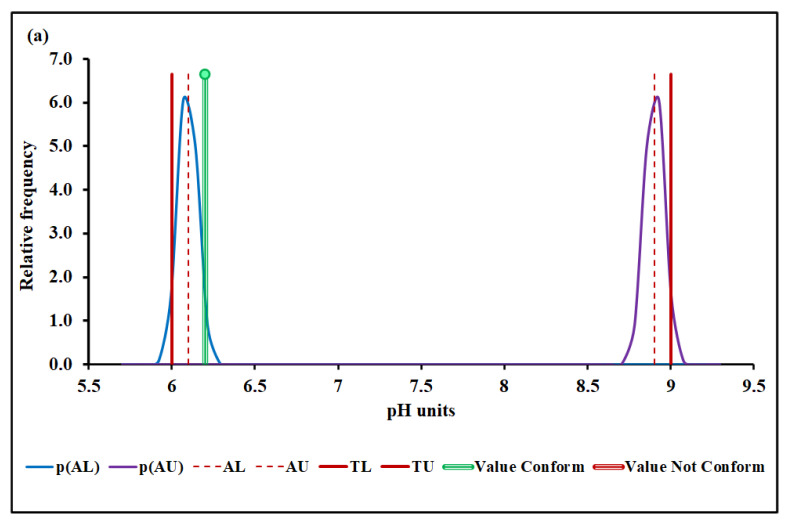

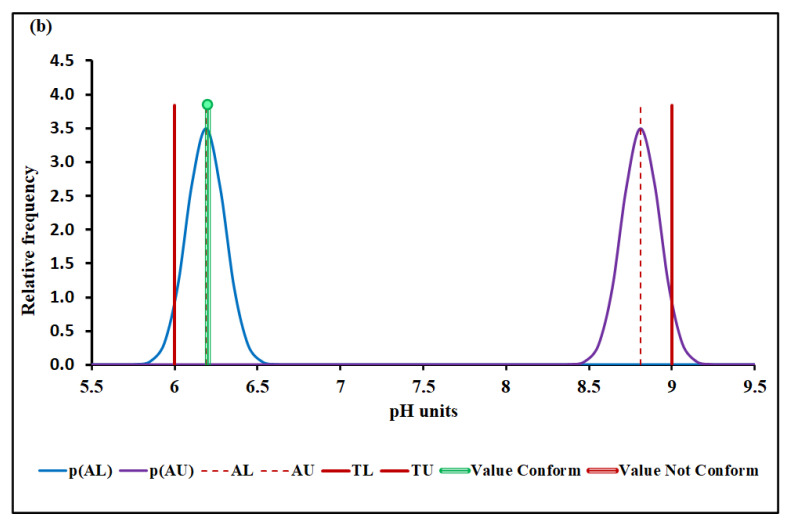
pH conformity assessment based on (**a**) MADe approach and (**b**) nIQR approach. p(AL)—probability density at the lower acceptance limit; p(AU)—probability density at the upper ac-ceptance limit; AL—lower acceptance limit; AU—upper acceptance limit; TL—lower tolerance limit; TU—upper tolerance limit.

Figure 3a,b show the pH conformity assessment based on MADe and nIQR, respectively.

The lower and the upper acceptance limits for pH were 6.10 and 8.90 (MADe approach) and 6.19 and 8.81 (nIQR approach), respectively.

#### 4.3.2. Biochemical Oxygen Demand (BOD)

**Figure 4:**
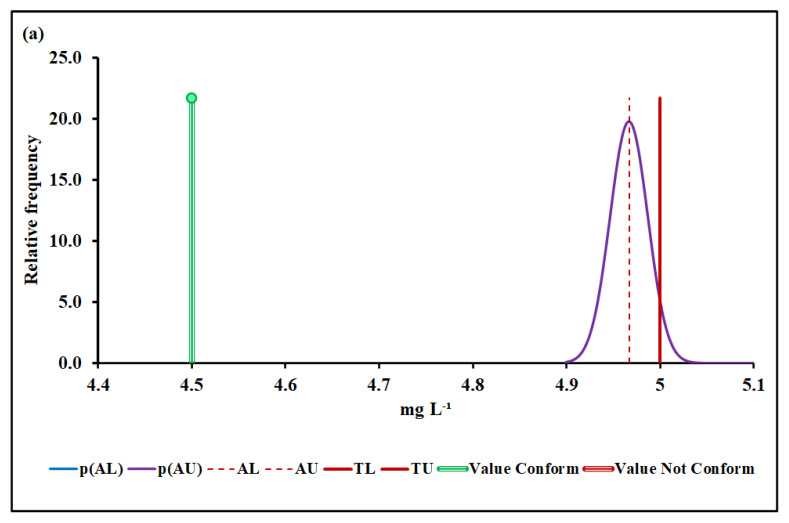

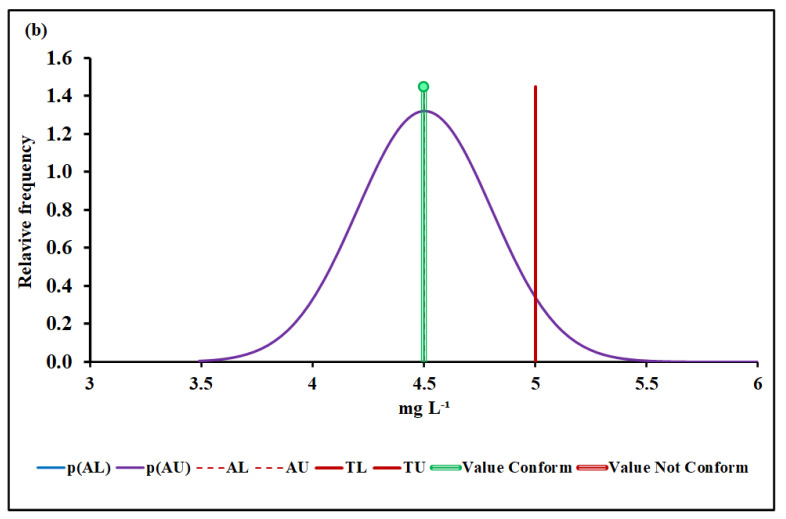
BOD conformity assessment based on (**a**) MADe approach and (**b**) nIQR approach. p(AL)—probability density at the lower acceptance limit; p(AU)—probability density at the upper acceptance limit; AL—lower acceptance limit; AU—upper acceptance limit; TL—lower tolerance limit; TU—upper tolerance limit.

Figure 4a,b show the BOD conformity assessment based on MADe and nIQR, respectively.

The upper acceptance limit for BOD was 4.42 mg L^−1^ (MADe approach) and 4.50 mg L^−1^ (nIQR approach), respectively.

#### 4.3.3. Manganese Molar Concentration

**Figure 5:**
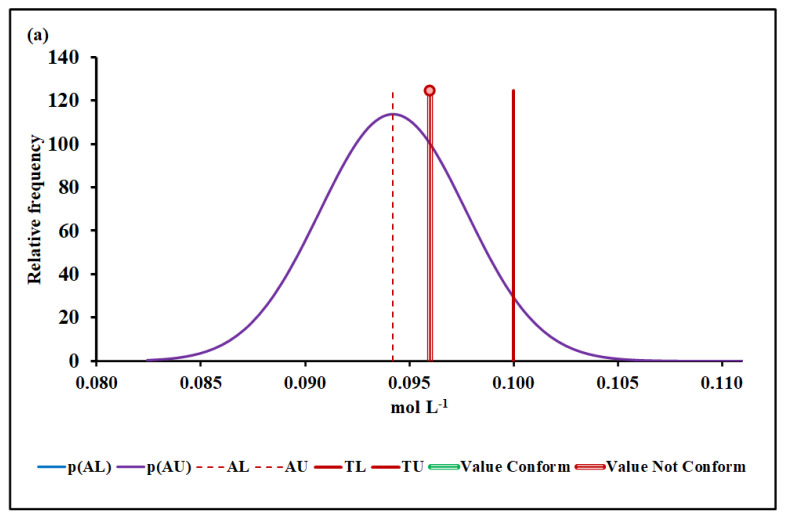

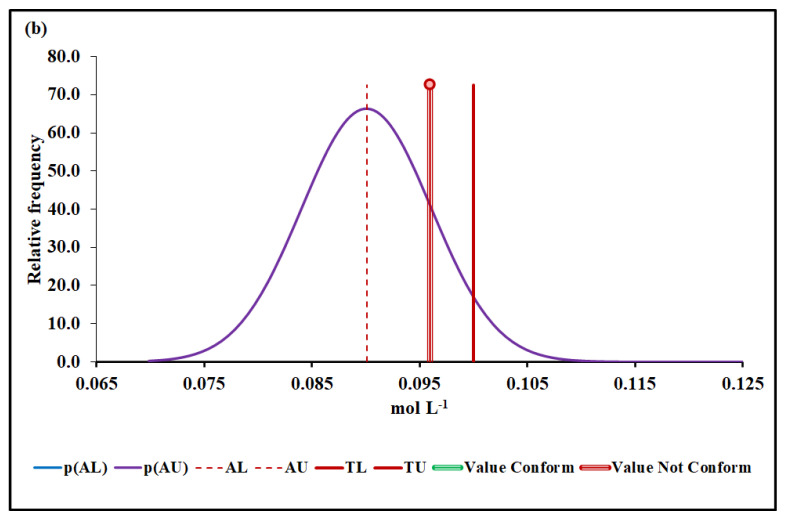
Mn molar concentration conformity assessment based on (**a**) MADe approach and (**b**) nIQR approach. p(AL)—probability density at the lower acceptance limit; p(AU)—probability density at the upper acceptance limit; AL—lower acceptance limit; AU—upper acceptance limit; TL—lower tolerance limit; TU—upper tolerance limit.

Figure 5a,b show the conformity assessment of the manganese molar concentration based on MADe and nIQR, respectively.

The upper acceptance limit for Mn was 0.094 mol L^−1^ (MADe approach) and 0.090 mol L^−1^ (nIQR approach), respectively.

#### 4.3.4. *Escherichia coli*

**Figure 6:**
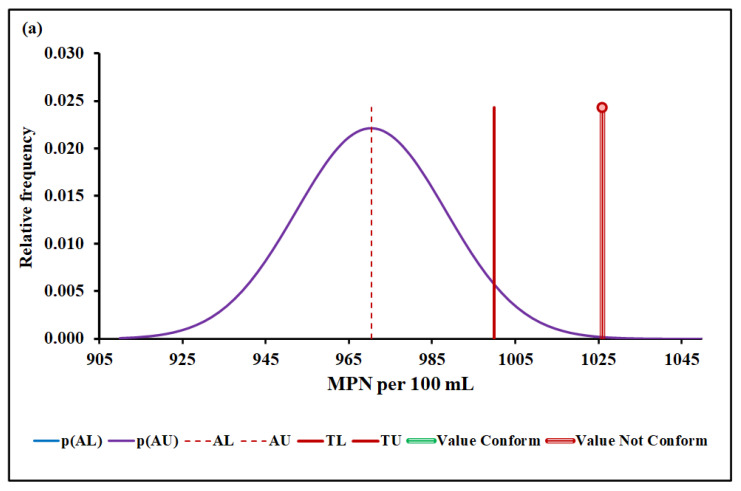

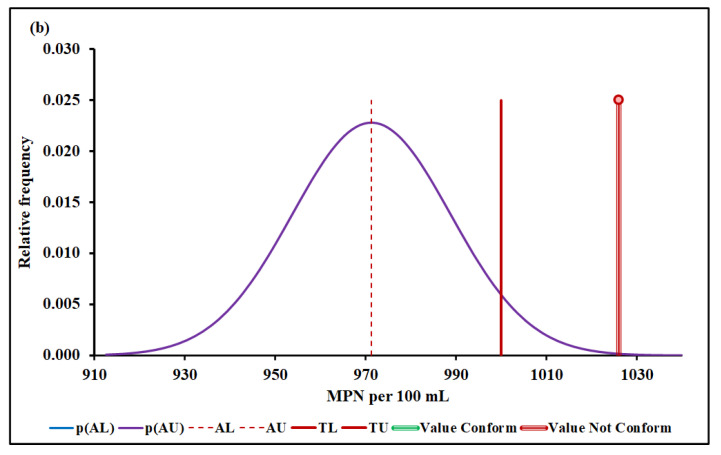
*Escherichia coli* conformity assessment based on (**a**) MADe approach and (**b**) nIQR approach. p(AL)—probability density at the lower acceptance limit; p(AU)—probability density at the upper acceptance limit; AL—lower acceptance limit; AU—upper acceptance limit; TL—lower tolerance limit; TU—upper tolerance limit.

Figure 6a,b show the *Escherichia coli* conformity assessment based on MADe and nIQR, respectively.

The upper acceptance limit for Escherichia coli was 970 MPN per 100 mL (MADe approach) and 971 MPN per 100 mL (nIQR approach), respectively.

#### 4.3.5. Summarizing Different Approaches

To prepare the discussion in the next section, Table 3 summarizes the comparison of the results for compliance assessment using the different methods (including the traditional approach, mean plus uncertainty arising standard deviation) and the percent consumer risk for each case.

## 5. Discussion

Some results showed partial overlaps between the central measures (medians) plus expanded uncertainties and their respective specification limits. It could generate doubts concerning the compliance assessment. Therefore, in this section, we discuss how our study could contribute to the solution of this problem. The robust methods were based on Equations (1)–(3), and the guard bands were calculated applying 1.64 $u\left(x^{*}\right)$.

Concerning the first property studied, pH, the conformity test has demonstrated, with a probability greater than 95% (significance level of 5%), that the pH, 6.20 (median), conforms to the requirement based on both MADe and nIQR approaches, even though the expanded uncertainty arising from nIQR approach is almost twice the MADe approach, in what was reflected in the risk for the consumer, although all of them were less than 5%. When the standard deviation approach was considered, no partial overlap was noticed, and the compliance assessment was clearly defined. For this biochemical parameter, all approaches presented agreed among themselves.

Regarding the second parameter, BOD, the conformity test has demonstrated, with a probability greater than 95% (significance level of 5%), that the BOD, 4.50 mg L^−1^ (median), conforms to the requirement based on both MADe and nIQR approaches, even though the expanded uncertainty arising from the nIQR approach is greater than the MADe approach, in what was reflected in the risk for the consumer, although both were less than 5%. When the standard deviation approach was considered, no partial overlap was noticed, and the non-compliance assessment was clearly defined. For this biochemical parameter, the nIQR approach seemed to be more appropriated because it was more conservative than the MADe approach; furthermore, the statistical methods were powerful enough to handle the absence of normality and the presence of outliers. It probably occurred because approximately 50% of the results were the same, so MADe tended to zero, not being a good estimator. Although 33% of BOD data are above the specification limit, they are not “lost”. These sample data estimate the population over time and apply robust methods to calculate the position measure (median), accompanied by its dispersion measures (MADe and nIQR) that are part of the entire data set. Here, we understand that the decision-making was based on all results during 2020 that generated a median value over time considering the heterogeneity of the data, and not just on a percentage of BOD data (33%).

For the next parameter, Mn, the conformity test has demonstrated, with a probability greater than 95% (significance level of 5%), that the manganese molar concentration, 0.096 mol L^−1^ (median), did not comply with the requirement based on both MADe and nIQR approaches. The expanded uncertainty arising from the nIQR approach is similar to the MADe approach; however, the risk for the consumer for nIQR approach is greater than the MADe approach. When the standard deviation approach was considered, no partial overlap was noticed, and the non-compliance assessment was clearly defined.

For the last property, *Escherichia coli*, the conformity test has demonstrated, with a probability greater than 95% (significance level of 5%), that *Escherichia coli*, 1026 MPN per 100 mL (median), does not conform to the requirement based on both MADe and nIQR approaches. Both the expanded uncertainty and the consumer’s risk arising from nIQR approach are similar to the MADe approach. On the other hand, no partial overlap was noticed when the standard deviation approach was considered, and the non-compliance assessment was clearly defined.

In the cases studied, the robust standard deviations calculated by MADe and nIQR approaches, which were used in the guard bands calculations, reached the same conclusions concerning compliance/non-compliance with the Brazilian specification, probably because the robustness of these estimators against outliers was not exceeded. Based on the robustness of statistical tests and knowledge about water analysis, consistent conclusions can be reached and supported by the expertise of the professional involved in this task. This study did not aim to make a decision based on a specific analysis or a fraction of samples to assess the compliance of river water analysis concerning Brazilian environmental regulations, but rather all the analyses carried out during 2020 considered the natural non-Gaussian behavior of the data and the median below/above of the specification limit, but with this limit within the uncertainty range.

## 6. Conclusions

This study aimed to use robust methods connected to the guard bands to assess the conformity when there is a partial overlap of the uncertainty bands about the specification limits of some biochemical parameters of the river water analysis. The methodology was considered suitable and promising since it respected the particularity of data sets.

The temporal heterogeneity of the samples was treated as a factor concerning the behavior of the samples and not as outliers. Moreover, robust methods connected to the concept of guard bands were beneficial for evaluating the risk of false conformity when there is a partial overlap between the most probable value accompanied by its uncertainty and the specification limit.

For future works, we propose applying this methodology to other water quality parameters and areas whose data are expected to have similar behavior, e.g., the classification of waste from steel companies and air quality. In addition, we recommend considering the spatial heterogeneity of the sampling target, as recently proposed by [47], and segregating the contribution from the sampling step and the analytical one.

## Figures and Tables

**Table 1:** Raw data of some biochemical properties of the Macaé River collected in 2020.

pH Results						BOD Results (mg L^−1^)					
7.20	5.10	5.23	6.30	6.23	5.90	4.40	18.00	4.50	4.50	4.50	3.97
5.99	6.01	6.00	6.10	5.80	6.10	4.40	4.50	4.50	101.00	4.50	3.97
5.86	5.86	5.91	5.93	5.96	6.00	91.91	4.50	4.50	4.50	6.23	4.18
6.12	6.19	6.20	6.21	6.24	6.29	4.20	92.30	77.85	4.50	4.50	4.18
6.09	6.19	6.02	6.73	6.12	6.86	4.60	4.50	4.50	39.18	4.50	5.72
6.97	7.05	7.08	7.23	7.30	7.53	4.80	4.50	4.50	4.50	4.50	5.72
6.30	7.50	6.30	6.30	6.57	6.34	4.40	4.50	4.50	4.50	5.91	5.91
5.90	8.10	6.06	6.11	6.10	6.20	4.50	4.50	4.50	4.50	4.50	4.50
6.93	6.94	7.66	7.65			7.80	4.50	83.25	22.37	2.33	6.23
						8.90	101.00	4.50	4.50	2.33	4.50
						10.00	4.50	98.97	4.50	3.48	6.92
						3.00	4.50	4.50	4.50	3.48	6.92
						6.98	83.25	8.76	91.91	4.50	4.50
						6.98	4.50	9.17	4.50	22.37	98.87
						8.76	4.10	9.17	92.30	4.50	4.50
						39.18	4.50	77.85	4.50		
Manganese results (mg L^−1^)						*Escherichia coli* results (MPN * per 100 mL)					
0.376	0.079	0.081	0.079	0.096	0.115	901	1138	914	500	1058	1043
0.091	0.270	0.092	0.030	0.096	0.115	1100	1131	1048	500	911	952
0.280	0.320	0.092	0.070	0.096	0.137	1126	1000	1042	970	1900	1019
0.081	0.290	0.099	0.210	0.096	0.137	1098	964	1023	1102	1034	1096
0.139	0.083	0.073	0.077	0.096	0.081	902	1220	1111	1042	1079	926
0.095	0.083	0.073	0.090	0.098	0.139	500	1220	1128	1056	983	950
0.310	0.083	0.300	0.230	0.100	0.140	901	800	998	1114	1110	1118
0.057	0.085	0.076	0.091	0.100	0.140	1000	1000	952	965	960	933
0.330	0.095	0.126	0.091	0.100	0.168	1028	1121	1036	991	1094	1030
0.012	0.091	0.078	0.092	0.100	0.168	1000	985	922	1100	1139	994
0.089	0.091	0.078	0.200	0.113	0.376						
0.079	0.081	0.099	0.096	0.113	0.080						

* Most Probable Number.

**Table 2:** Statistical results.

	pH	BOD (mg L^−1^)	Mn (mol L^−1^)	*Escherichia coli* (MPN per 100 mL)
Specification limit ‡	6–9	5 ^†^	0.1 ^†^	1000^†^
Median	6.20	4.5	0.0956	1026
Mean	6.40	17.2	0.128	1016
Standard deviation (SD)	0.62	29.1	0.081	184
IQR	0.870	3.095	0.0553	142
$s^{*}\mathrm{nIQR}$	0.645	2.2943	0.0410	105
$s^{*}\mathrm{MADe}$	0.334	0.1483	0.0248	109
*p*	52	94	72	60
U(x) SD	0.18	6.1	0.019	48
$U\left(x^{*}\right)$ nIQR	0.23	0.60	0.012	35
$U\left(x^{*}\right)$ MADe	0.12	0.04	0.007	36
$g=1.64\times 1.25\times \frac{s^{*}\mathrm{nIQR}}{\sqrt{p}}$	0.18	0.48	0.010	28
$g=1.64\times 1.25\times \frac{s^{*}\mathrm{MADe}}{\sqrt{p}}$	0.095	0.03	0.006	29

^†^ Upper specification limit, ‡ based on Brazilian regulations.

**Table 3:** Summarizing and comparing different approaches.

	pH		BOD (mg L^−1^)		Mn (mol L^−1^)		*Escherichia coli* (MPN per 100 mL)	
	Result	Consumer’s Risk	Result	Consumer’s Risk	Result	Consumer’s Risk	Result	Consumer’s Risk
Median and $U\left(x^{*}\right)$ MADe	6.20 ± 0.12	0.04%	4.50 ± 0.04	0.00%	0.096 ± 0.007	12.7%	1026 ± 36	7.4%
Median and $U\left(x^{*}\right)$ nIQR	6.20 ± 0.23	4.0%	4.50 ± 0.60	4.9%	0.096 ± 0.012	25.3%	1026 ± 35	6.9%
Mean and U(x) SD	6.40 ± 0.18	0.00%	17.2 ± 6.1	Without overlap	0.128 ± 0.019	Without overlap	1016 ± 48	25.2%

## Data Availability

Restrictions apply to the availability of these data. Data was obtained from the Telos Soluções Ambientais and are available from the authors with the permission of them.
